# Coronavirus Interplay With Lipid Rafts and Autophagy Unveils Promising Therapeutic Targets

**DOI:** 10.3389/fmicb.2020.01821

**Published:** 2020-08-11

**Authors:** Katia Fecchi, Simona Anticoli, Daniela Peruzzu, Elisabetta Iessi, Maria Cristina Gagliardi, Paola Matarrese, Anna Ruggieri

**Affiliations:** Reference Center for Gender Specific Medicine, Istituto Superiore di Sanità, Rome, Italy

**Keywords:** coronavirus, lipid rafts, autophagy, SARS-CoV-2, drugs

## Abstract

Coronaviruses are enveloped, single-stranded, positive-sense RNA viruses that can infect animal and human hosts. The infection induces mild or sometimes severe acute respiratory diseases. Nowadays, the appearance of a new, highly pathogenic and lethal coronavirus variant, SARS-CoV-2, responsible for a pandemic (COVID-19), represents a global problem for human health. Unfortunately, only limited approaches are available to treat coronavirus infections and a vaccine against this new coronavirus variant is not yet available. The plasma membrane microdomain lipid rafts have been found by researchers to be involved in the replication cycle of numerous viruses, including coronaviruses. Indeed, some pathogen recognition receptors for coronaviruses as for other viruses cluster into lipid rafts, and it is therefore conceivable that the first contact between virus and host cells occurs into these specialized regions, representing a port of cell entry for viruses. Recent data highlighted the peculiar pro-viral or anti-viral role played by autophagy in the host immune responses to viral infections. Coronaviruses, like other viruses, were reported to be able to exploit the autophagic machinery to increase their replication or to inhibit the degradation of viral products. Agents known to disrupt lipid rafts, such as metil-β-cyclodextrins or statins, as well as autophagy inhibitor agents, were shown to have an anti-viral role. In this review, we briefly describe the involvement of lipid rafts and autophagy in coronavirus infection and replication. We also hint how lipid rafts and autophagy may represent a potential therapeutic target to be investigated for the treatment of coronavirus infections.

## Introduction

Coronaviruses (CoVs) are a large group of enveloped animal and human viruses, with a single-stranded positive-sense RNA genome. The Coronaviridae family, to which they belong, includes four genera of CoVs, indicated as Alphacoronavirus (αCoV), Betacoronavirus (βCoV), Gammacoronavirus (γCoV), and Deltacoronavirus (δCoV) (Cui et al., 2019; Chen et al., 2020). αCoV and βCoV have evolutionary evolved from bats and rodents, and have been responsible for human infections; whereas δCoV and γCoV derive from avian species (Woo et al., 2012). αCoV and βCoV are further divided into subgroups, 1a-1b and 2a-2d, respectively, (Graham et al., 2013; Drexler et al., 2014; Cui et al., 2019; Fung and Liu, 2019) (Supplementary Figure S1). Coronaviruses have been regarded as etiologic agents of both relatively mild and severe respiratory infections/diseases in humans (Zhao et al., 2012; Paules et al., 2020). HCoV-NL63, HCoV-229E, HCoV-OC43, and HCoV-HKU1 cause the common cold and mild upper respiratory diseases in immunocompetent hosts, although some of them can be potentially virulent in infants, young children, and elder individuals. Highly pathogenic human coronaviruses, responsible for more severe respiratory diseases, include SARS-CoV that caused the 2003 pandemic outbreak of severe respiratory tract infection (Ksiazek et al., 2003), MERS-CoV, responsible for the 2012 outbreak in Middle Eastern countries (Zaki et al., 2012), and the novel SARS-CoV-2, actually causing a pandemic of severe pneumonia that started in China in December 2019 (Benvenuto et al., 2019; Zhu et al., 2020).

Coronavirus and coronavirus-like infections have been described also in domestic and wild animals, such as swine [porcine transmissible gastroenteritis virus and porcine epidemic diarrhea virus], cattle (BCoV), horses, camels, cats, dogs, rodents, birds, bats, rabbits, ferrets, mink, and various wildlife species (Fehr and Perlman, 2015; Lin et al., 2016; Banerjee et al., 2019; Cui et al., 2019; Ye et al., 2020). Although many coronavirus infections are subclinical in animals, however, they cause a range of diseases that can have serious consequences for animal health as well as for the economic losses in livestock. In addition, domestic animals may have been important intermediate hosts for virus transmission from natural hosts to humans (Fehr and Perlman, 2015; Ye et al., 2020).

Since only limited approaches are available to treat or prevent coronavirus infections (Zhang et al., 2020), the importance of identifying novel therapeutic targets is evident.

Viruses have evolved a close interplay with the host cell and the first encounter between the virus and target cell occurs at the plasma membrane. Lipid rafts are specialized plasma membrane microdomains involved in important processes of the virus infections and of the host target cells (Rosenberger et al., 2000).

Viruses exploit lipid rafts to gain infection of the target cells; therefore, pharmacologic depletion or disruption of lipid rafts may provide a tool to reduce or inhibit viral replication (Khandia et al., 2019).

Furthermore, it has been widely demonstrated that lipid rafts play a fundamental role in autophagic machinery: they are associated with autophagosome morphogenesis, either in the initiation or in the maturation phases (Matarrese et al., 2014).

Autophagy, a cell process involved in cellular homeostasis, stress, and immune responses to viral infections, is a two-edged process in virus infections, since it may have pro- or anti-viral roles (Kudchodkar and Levine, 2009; Khandia et al., 2019). Viruses, on the other side, during co-evolution with the host cell, developed mechanisms to usurp/exploit the host autophagic system (Jheng et al., 2014).

This minireview reports on the available knowledge about the interplay between coronaviruses, including the SARS-CoV-2, with lipid rafts and autophagic pathways, in order to focus the attention to novel potential targets to inhibit coronavirus infections.

### Lipid Rafts and Autophagy: Focus on Coronavirus

Lipid rafts are plasma membrane microdomains (10–200 nm) enriched in cholesterol, glycosphingolipids, and phospholipids. They are also found in the endoplasmic reticulum (ER) (Browman et al., 2006), in the Golgi complex (Eberle et al., 2002), and on the membrane of endosomes (Sobo et al., 2007) and phagosomes (Dermine et al., 2001). There are two main types of lipid rafts based on their protein composition: “planar lipid rafts” and “caveolae” enriched by the proteins flotillin and caveolin, respectively (McGuinn and Mahoney, 2014). Both possess a similar lipid composition that confers resistance to solubilization by non-ionic detergents at low temperatures (Brown and Rose, 1992), and the characteristic of being able to be isolated in sucrose gradient (Sargiacomo et al., 1993). Because of their dynamic and heterogeneous structure, they can rapidly assemble and disassemble, changing their composition in response to intra- and extracellular stimuli (Simons and Toomre, 2000). A key component of lipid rafts is cholesterol that is the glue that maintains raft architecture, as demonstrated by the disorganization and disruption of lipid rafts upon depletion of plasma membrane cholesterol by the cholesterol depleting agent methyl-β-cyclodextrin (MβCD) (Simons and Ehehalt, 2002; Zidovetzki and Levitan, 2007). These membrane regions play an important role in a variety of cellular functions, but principally they recruit and concentrate several signaling molecules (Song et al., 1997; Galbiati et al., 1999; Razani et al., 1999; Patel and Insel, 2009) that, by interacting with caveolin and flotillin, form a sort of signal transduction platform. Therefore, lipid rafts are involved in many biological functions including endocytosis, signal transduction, cell communication, and regulation of autophagy (Nabi and Le, 2003; Shi et al., 2015).

Because of their capacity to cluster into a “phagocytic synapse” several pathogen recognition receptors (Toll-like receptors, C-type lectin receptors), lipid rafts are the focus of intense research in the field of infection. In particular, they are involved in several steps along a viral infection, such as virus entry into the host cell (fusion and internalization), viral protein transport, viral assembly, and budding processes (Hogue et al., 2012). Several enveloped (HIV-1, influenza viruses, coronavirus, flavivirus) and non-enveloped (SV40 and Rrotavirus) viruses exploit the raft platform to bind to their specific receptors (Ono and Freed, 2001; Pelkmans, 2005; Li et al., 2007; Takahashi and Suzuki, 2011).

**FIGURE 1:**
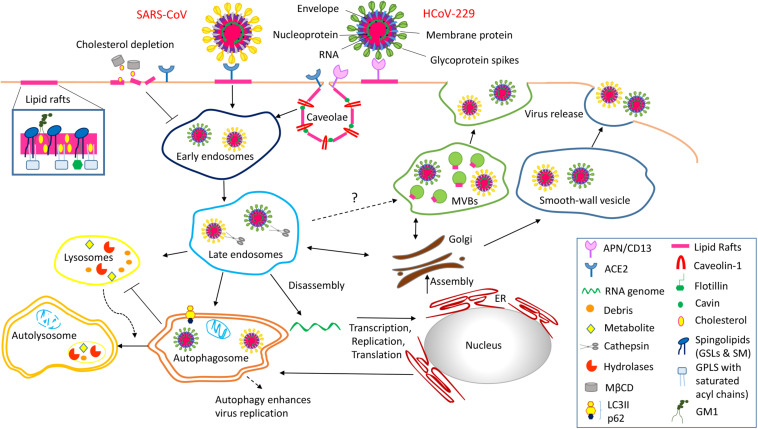
Schematic representation of the role of lipid rafts in Coronavirus infection of the host cells is a multistep endocytic process characterized by a series of complex events tightly regulated in space and time. Step 1 Entry process of coronavirus into the host cells is initiated by the binding of the spike glycoprotein with the specific receptor (ACE2, APN/CD13) located into lipid rafts/caveolae. This interaction causes conformational changes of the viral particle, which trigger specific signaling events necessary for the viral entry mechanism. Step 2 Lipid rafts/caveolae-mediated endocytosis is followed by intracellular trafficking of virus particles in transport vesicles (early and late endosomes). The low pH in late endosomes induces a conformational change in coronavirus that mediates fusion of the viral envelope with the endosomal membrane. Step 3 Viral genomes are translated in two polyproteins, pp1a and pp1ab, which encode the non-structural viral proteins that form the replication transcription complex. This complex produces genomic RNA as well as multiple subgenomic mRNAs encoding structural proteins. Translation of mRNA encoding for the nucleocapsid proteins occurs in the cytoplasm where the newly synthesized proteins interact with new genomes to form ribonucleoprotein particles. In contrast, matrix, envelope and spike proteins translation occurs into the ER. Coronavirus uses also the autophagy machinery for replication and has evolved strategies to avoid autophagy-induced lysosomal degradation. Step 4 After assembly the progeny viral particles, virus-containing vesicles (smooth-wall vesicles) are budded and released into the extracellular environment through fusion with the plasma membrane (exocytosis). Alternatively, we speculate that coronavirus might utilize multivescicular bodies (MVBs) and take advantage of the exosomal pathway for egress.

Recent studies have implicated lipid rafts in coronavirus entry and egress through a multistep endocytic process, although the detailed mechanism remains to be disclosed (Li et al., 2007). Figure 1 depicts a schematic view of the role of lipid rafts in two paradigmatic human coronaviruses, HCoV-229E and SARS-CoV, infections. Briefly, the receptors for the two coronaviruses, aminopeptidase N (APN/CD13) and angiotensin converting enzyme 2 (ACE2), respectively, are located into lipid rafts and play a fundamental role in the initial step of the virus infection. APN/CD13, a zinc-binding aminopeptidase, is expressed in several cell types (endothelial, granulocytic, and monocytic cells; epithelial cells of the kidney; respiratory tract and intestine) and is required also for porcine, canine, and feline coronavirus recognition. ACE2, encoded on the X chromosome, is a metalloprotease long known to be a key player in the renin–angiotensin system that co-localizes with caveolin-1 and GM1 and is expressed on type I and II pneumocytes, enterocytes, endothelial cells of the heart and kidney, epithelial cells of the kidney, and the testis (Hamming et al., 2004). SARS-CoV-2, the etiological agent of the COVID-19 pandemic, also binds ACE2 to infect host cells, by similarity to SARS-CoV, from which it differentiates for point mutations on the spike protein, essential for receptor binding (Wang et al., 2020; Yan et al., 2020). By analogy to SARS-CoV, it can be hypothesized that SARS-CoV-2 may also use lipid rafts for its entry into the host cells.

The steps following coronavirus entry are not clearly identified. A recent study demonstrated that SARS-CoV exploits the activity of cathepsin L, an endosomal cysteine protease, to initiate proteolysis and activation of membrane fusion within endosomes (Simmons et al., 2013). In addition, coronavirus mouse hepatitis virus (MHV) infection is inhibited when early endosome-associated proteins, RAB5 and EEA1, are down-regulated by siRNA (Burkard et al., 2014) and infectious bronchitis virus (IBV) release of the nucleocapsid into the cytoplasm (Wang et al., 2018) occurs through the fusion with endosome–lysosome membranes. These data suggest that coronaviruses exploit the early and late endosome compartment after viral entry, as several other viruses (adenovirus, human papillomavirus, polyomavirus, African swine fever virus, and influenza virus) (Lakadamyali et al., 2004; Sánchez et al., 2017; Spriggs et al., 2019).

Recent evidence shows that there is a close interplay between lipid rafts and autophagy during physiological cell function, as lipid rafts can regulate autophagy by interacting with autophagosomes and autophagy-related proteins, such as ATG5 and ATG12 (Chen et al., 2014; Garofalo et al., 2016). In addition a functional and structural link between lipid rafts and autophagy comes from the evidence that chemical (e.g., MβCD, fumonisin B1) or biological (e.g., siRNA inhibition of key enzymes involved in the sphingolipids metabolism) molecules capable of altering the chemical composition or molecular organization of lipid rafts have also a strong impact on autophagy (Khandia et al., 2019).

Autophagy is an evolutionarily conserved (from yeast to mammals), selective, and finely regulated process that generates ATP and precursors for the synthesis of macromolecules (Ktistakis and Tooze, 2016) and is considered as a survival process put in place by cells under stressful conditions, such as pathogen microbial infections. It plays a key role in cellular homeostasis and is responsible for the turnover of cellular organelles (Yu et al., 2018), as its main function is to remove and recycle non-essential, damaged, or obsolete cellular components, such as whole organelles or macromolecules (Mizushima and Komatsu, 2011). During autophagy, cytoplasmic portions are enclosed into double membrane bound vesicles (autophagosomes) that then fuse with late endosomes/lysosomes, whose contents are degraded by lysosomal proteases. Autophagy has been shown to have additional function in innate immunity, by degradation of viruses, or intracellular pathogens, as well as by presenting pathogen components to the immune system (Mao et al., 2019).

Autophagy can have a pro-viral role, promoting virus infection and propagation, as well as an antiviral role, essentially depending on the virus strain, on the phase of infection, on the infected cell type, but also on the cellular microenvironment (Lennemann and Coyne, 2015). During co-evolution with their natural hosts, viruses developed the ability to hijack autophagic mechanisms to their advantage by using them for immune escaping, or using autophagosomes as a replicative niche. However, data on autophagy interplay with coronaviruses are scant so far, related on no more than 50 works published between 2004 and 2015. In general, it is suggested that CoVs can interact with some components of the autophagic pathway in an opposite way with a dual effect: utilizing autophagy components to promote viral replication and/or to inhibit degradation of viral products through the autophagic pathway (Cong et al., 2017). In some coronaviruses, infection results are still contradictory, as in the case of MERS-CoV (Corman et al., 2016), which has been reported to induce phosphorylation changes in key kinases, such as AKT1 and mTOR, that regulate the early steps of autophagic process (Kindrachuk et al., 2015), thus stimulating autophagy in infected cells. In contrast, Gassen et al. reported that MERS-CoV activates the SKP2 kinase, consequently reducing Beclin1 (BECN1) activity and inhibiting the fusion of autophagosomes with lysosomes, which results in autophagy inhibition (Gassen et al., 2019).

In general, it has been hypothesized that coronaviruses would induce accumulation of autophagic vacuoles to obtain a larger availability of the membrane structure necessary for their replication (Gassen et al., 2019), which is consistent to the observed co-localization of the rodent coronavirus, MHV, replication complex, with the autophagic proteins LC3 and Apg12 (Prentice et al., 2004). Likewise, the SARS-CoV takes advantage of autophagic pathway to replicate and transcribe its own genome, as suggested by inhibition of its replication through inactivation of GSK-3 (glycogen synthase kinase-3), a serine/threonine kinase that inhibits autophagy, through the mammalian target of rapamycin (mTOR) complex 1 (mTORC1) (Wu et al., 2009; Zúñiga et al., 2010). A very recent study showed that SARS-CoV-2, similar to MERS-CoV, strongly reduced the autophagic flux in infected cell lines downregulating the AMPK/mTORC1 pathway, altered autophagy-relevant signaling, and also reduced autophagosome/lysosome fusion efficiency (Gassen et al., 2020). As a result, the SARS-CoV-2 virus could take advantage of the autophagy reduction, thus preventing viral product degradation and enhancing double membrane vesicles (DMV) availability, indispensable for their replication.

The cell organelle most involved in the dynamic membrane changes is the ER. It is therefore not surprising that some coronaviruses, through the insertion of their trans-membrane glycoproteins at the ER level, are able to induce ER stress. This is the case, for example, of the ORF3 protein produced by porcine epidemic diarrhea virus (PEDV), which induced ER stress-dependent autophagy in different porcine and human cell types (Zou et al., 2019). In the same vein, MHV was observed to use the host machinery to export vesicular ER to originate membranes for the genesis of DMV. As regards the non-structural protein (NSP) 6 of the avian infectious bronchitis virus (IBV), a gamma-coronavirus, in addition to providing membranes useful for the replication of the virus, it could prevent its degradation within the lysosomes, effectively escaping the autophagy-based cellular defensive mechanisms. Similar properties have also been observed for MHV and SARS NSP6 proteins and PRRSV arterivirus NSP5, NSP6, and NSP7 (Cottam et al., 2014).

## Discussion

Coronaviruses may represent a threat for human and animal health for their potential to cross the species barrier, thus acquiring human virulence, as evidenced by the previous SARS-CoV and MERS-CoV outbreaks and by the ongoing COVID-19 pandemic. Notwithstanding, treatment and prophylaxis measures to control coronavirus infections are lacking so far, either in humans or in livestock animals.

As outlined in this review, lipid rafts and autophagic pathways play a pivotal role in coronavirus infection, being critical for viral entry and replication, as well as for viral release from the host cells. Actually, lipid rafts are the focus of intense research in the field of infection, and it is conceivable to consider targeting some lipid raft components in order to inhibit virus infection at the cell level.

In particular, lipid raft disruption by cholesterol-depleting agents has been shown to inhibit infection of several microbes by blocking their entry into the host cells (Nomura et al., 2004; Thorp and Gallagher, 2004; Choi et al., 2005; Riethmüller et al., 2006; Glende et al., 2008; Lu et al., 2008; Guo et al., 2017; Owczarek et al., 2018; Szczepanski et al., 2018; Abu-Farha et al., 2020; Baglivo et al., 2020; Fantini et al., 2020; Rodrigues-Diez et al., 2020; Table 1). In human immunodeficiency virus (Ono and Freed, 2001), herpes simplex virus, and rotavirus infections, MβCD, a widely used raft-disrupting agent, has been shown to affect virus entry, thereby reducing their infectivity (Dou et al., 2018; Wudiri et al., 2017). In addition, in Japanese encephalitis virus and dengue virus infection, disruption of lipid rafts by MβCD has been shown to decrease viral infection acting at both viral entry and intracellular replication step (Lee et al., 2008). β-cyclodextrins are extensively utilized in pharmaceutical formulations as excipients to enhance solubility, bioavailability, and stability of many drugs (Loftsson and Brewster, 2012). In the light of the above-described antiviral activity, further studies on pharmacokinetic and safety of MβCD could foster its clinical application as an antimicrobial agent in humans. Statins, used in human therapy for their ability to inhibit cellular synthesis of cholesterol, have also been reported to have an anti-viral effect, inhibiting infection of flavivirus, such as dengue virus (DENV), hepatitis C virus (HCV), West Nile virus (WNV), and Zika virus (Mackenzie et al., 2007; Andrus and East, 2010; Martínez-Gutierrez et al., 2011; Españo et al., 2019), and their application to inhibition of the coronavirus infection would be worth considering.

**TABLE 1:** Function of lipid rafts and autophagy in coronavirus lifecycles: overview on drugs.

Drugs	Mechanism of action	Target virus	Antiviral effects	References
MβCD, Nystatin	Disrupt lipid raft architecture by depleting cell membrane cholesterol	CRCoV HCoV-229E HCoV-NL63 HCoV-OC43 IBV MERS-CoV MHV SARS-CoV-2	Inhibit viral entry/endocytosis	Nomura et al., 2004; Thorp and Gallagher, 2004; Choi et al., 2005; Glende et al., 2008; Lu et al., 2008; Guo et al., 2017; Owczarek et al., 2018; Szczepanski et al., 2018; Baglivo et al., 2020; Fantini et al., 2020
Statins (mevastatin)	Inhibit lipid raft formation by lowering cholesterol biosynthesis	IBV SARS-CoV-2	Inhibit viral entry/endocytosis	Guo et al., 2017; Abu-Farha et al., 2020; Rodrigues-Diez et al., 2020
Chloroquine and Hydroxychloroquine	Interfere with lysosome-mediated autophagy function by increasing the endosomal/lysosomal pH	MERS-CoV HCoV-229E HCoV-OC43 SARS-CoV SARS-CoV-2	Prevent viral fusion and inhibit the viral entry by endocytosis, uncoating and exit (exocytosis) process	ClinicalTrials.gov, 2020a,b,c,d,e,f,g,h,i,j
Rapamycin (Sirolimus)	Inhibits mammalian target of rapamycin (mTOR) kinase	MERS-CoV PEDV SARS-CoV-2	Downregulates virus infection	ClinicalTrials.gov, 2020k
Nitazoxanide	Inhibits Akt/mTOR/ULK1 signaling pathway	HCoVOC43 MERS-CoV SARS-CoV-2	Inhibits virus replication	Shakya et al., 2018; Yang et al., 2020
Niclosamide	mTORC1 inhibitor	MERS-CoV SARS-CoV	Inhibits viral antigen synthesis	Liu et al., 2019; Xu et al., 2020

Likewise, the pharmacological modulation of autophagic processes can represent an attractive therapeutic strategy for elimination of the viral pathogen or containment of the infection (Rubinsztein et al., 2009; Clark et al., 2018).

In fact, different drugs described as inhibitors or inducers of the autophagy that control host cell pathways process involved in coronavirus infection, have sparked interest for their potential antiviral activity (Shakya et al., 2018; Liu et al., 2019; Xu et al., 2020; Yang et al., 2020; Table 1). One of this, chloroquine (CQ), and its derivative hydroxychloroquine (HCQ), known as anti-malarial drugs (O’Neill et al., 1998), which are able to inhibit autophagy by raising the lysosomal pH (Golden et al., 2015), have also been evaluated for HIV infection (Romanelli et al., 2004). Very recently, clinicians have paid attention to CQ and HCQ as a possible treatment of patients infected by the novel emerged SARS-CoV-2 (ClinicalTrials.gov, 2020a,b,c,d,e,f,g,h,i,j). This insight is also supported by some recent works, including a recent publication by Gao et al. that indicates some positive effects of CQ on the course of pneumonia associated with the infection and on the reduction in healing (Cortegiani et al., 2020; Gao et al., 2020; Geleris et al., 2020; Yu et al., 2020).

On the contrary, other studies have highlighted the ineffectiveness of these drugs both in the viremic phase and in respiratory complications (Boulware et al., 2020; Mahévas et al., 2020; Rosenberg et al., 2020). Although some data, both referring to COVID-19 and SARS, have highlighted some positive effects of CQ and HCQ in the evolution of the disease, thus showing their therapeutic potential, at the moment, there are no data that can support the use of these drugs to control the entire pathological process related to SARS-CoV-2 (Gies et al., 2020).

In the same vein, rapamycin, also known as sirolimus, an autophagy inducer already used as an immunosuppressant, has been tested, with some success, in the treatment of COVID-19 (NCT04341675) (ClinicalTrials.gov, 2020k). These cases may represent a repositioning of the drugs with clinical success in treatment areas beyond their original approved use. According to this, the antiviral *in vitro* activity of spermidine, niclosamide, and nitazoxanide (known autophagy inducers) vs. SARS-CoV-2 was recently reported (Shakya et al., 2018; Liu et al., 2019; Xu et al., 2020; Yang et al., 2020). Thus, a prophylactic approach to COVID-19 using these drugs, which are well tolerated, clinically applied, or FDA-approved compounds, would be rational (Gassen et al., 2020). Importantly, treatments for emerging infections by targeting host cell pathways, rather than the infectious agent directly, or to complement antivirals with drugs that enhance host cell resistance mechanisms have thus become an active and promising therapeutic strategy. This strategy is even more important and urgent to be explored in the case of such potentially and suddenly pandemic virus family, as coronaviruses are.

## Author Contributions

KF contributed to the design of this study, drafted the manuscript, and drew figures relative to lipid rafts. SA contributed to the conception of the idea and drafted the manuscript and figure relative to coronavirus description. DP conducted a wide literature search and drafted the manuscript on the lipid rafts section. EI drafted the manuscript on the autophagy section. PM provided autophagy expertise, contributed to draft and revision of the manuscript, and concurred with the final version of the review. MG provided lipid rafts expertise, and contributed to draft and review the manuscript and to the organization of the review. AR conceived the idea, drafted and revised the manuscript, coordinated the activity, and concurred with the final version of the review. All authors contributed to the article and approved the submitted version.

## Conflict of Interest

The authors declare that the research was conducted in the absence of any commercial or financial relationships that could be construed as a potential conflict of interest.

## Supplementary Material

FIGURE S1 Taxonomy of CoVs Classification scheme of some representative human (in red) and animal (in black) coronaviruses.Abbreviations: FCoV, feline coronavirus; TGEV, transmissible gastroenteritis virus; PRCV, porcine respiratory coronavirus; HCoV, human coronavirus; PEDV, porcine epidemic diarrhea virus; Ro-BatCoV, Rousettus bat CoV; PHEV, porcine hemagglutinating encephalomyelitis virus; MHV, mouse hepatitis virus; BCV, bovine coronavirus; SARS-CoV, severe acute respiratory syndrome coronavirus; Bat SARSr-CoV, Bat SARS-like coronavirus; MERS-CoV, Middle East respiratory syndrome coronavirus; BuCoV, bulbul coronavirus; PDCoV, porcine deltacoronavirus; BWCoV, beluga whale coronavirus; IBV, avian infectious bronchitis virus; TCoV, Turkey coronavirus.
