# Supplementary figures and images for: Coronavirus Interplay With Lipid Rafts and Autophagy Unveils Promising Therapeutic Targets
Source: Front Microbiol. 2020 Aug 11;11:1821. doi: 10.3389/fmicb.2020.01821 (PMC7431668; doi:10.3389/fmicb.2020.01821)

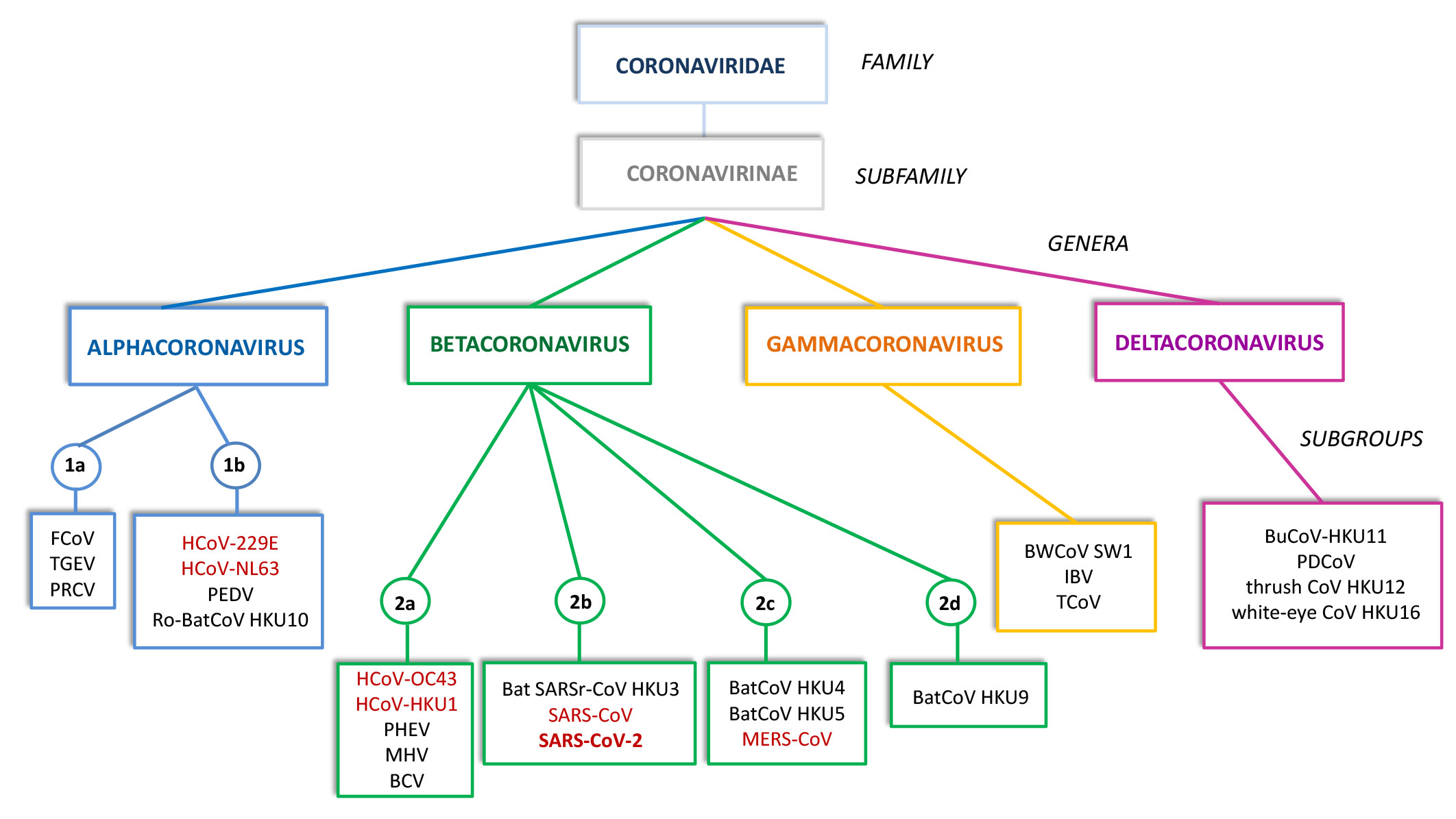

Supplement: FIGURE S1 — Taxonomy of CoVs Classification scheme of some representative human (in red) and animal (in black) coronaviruses.Abbreviations: FCoV, feline coronavirus; TGEV, transmissible gastroenteritis virus; PRCV, porcine respiratory coronavirus; HCoV, human coronavirus; PEDV, porcine epidemic diarrhea virus; Ro-BatCoV, Rousettus bat CoV; PHEV, porcine hemagglutinating encephalomyelitis virus; MHV, mouse hepatitis virus; BCV, bovine coronavirus; SARS-CoV, severe acute respiratory syndrome coronavirus; Bat SARSr-CoV, Bat SARS-like coronavirus; MERS-CoV, Middle East respiratory syndrome coronavirus; BuCoV, bulbul coronavirus; PDCoV, porcine deltacoronavirus; BWCoV, beluga whale coronavirus; IBV, avian infectious bronchitis virus; TCoV, Turkey coronavirus. [file Image_1.TIF]
